# Multiomics‐Driven Drug‐Cell Interaction Network for Chemotherapy Sensitivity Prediction in Metabolically Defined Triple‐Negative Breast Cancer Subtypes

**DOI:** 10.1111/jcmm.70572

**Published:** 2025-06-02

**Authors:** Jingyuan Zhang, Xuejun Sun

**Affiliations:** ^1^ Department of Breast Surgery Shannxi Provincial Cancer Hospital Xi'an Shaanxi China; ^2^ Department of General Surgery The First Affiliated Hospital of Xi'an Jiaotong University Xi'an Shaanxi China; ^3^ Department of Radiation Oncology The First Affiliated Hospital of Xi'an Jiaotong University Xi'an Shaanxi China

**Keywords:** drug efficacy prediction, metabolic heterogeneity, multi‐omics, triple‐negative breast cancer

## Abstract

Triple‐negative breast cancer (TNBC) is associated with a poor prognosis due to insufficient molecular subtyping precision and limited actionable targets. Although metabolic reprogramming underlies TNBC chemotherapy resistance, establishing metabolic subtyping systems and investigating drug sensitivity across distinct metabolic subgroups could provide novel therapeutic avenues for breast cancer management. GSVA (Gene Set Variation Analysis) analysis of metabolic pathways reveals significant differences in TNBC (Triple‐Negative Breast Cancer) patients. TNBC patients are classified into four metabolic subtypes through consensus clustering, based on their GSVA values of metabolic pathways. These subtypes are: MS_1, characterised by increased lipogenic activity; MS_2, characterised by increased carbohydrate and nucleotide metabolism; MS_3, a metabolism‐active subtype with activation of all types of metabolism; and MS_4, characterised by suppressed metabolic activity across all types of metabolism. We next propose a novel method called MODIN (Multiomics‐Driven Drug‐Cell Interaction Network), which embeds multi‐omics gene information (mRNA expression, copy number variation and DNA methylation) and drug SMILES data into a latent space, and then employs a multi‐head attention‐based interaction module to accurately predict the LN_IC50 values of 621 drugs in TNBC. Based on MODIN, noteworthy disparities in drug sensitivity emerge between the patient cohorts categorised as MS_2 and MS_3. MS_3 patients show a significantly higher sensitivity to chemotherapy regimens, especially for doxorubicin and docetaxel, while the MS_2 cohort displays marked resistance to these drugs. Our study reveals the metabolic heterogeneity of TNBC, and TNBC patients with increased carbohydrate and nucleotide metabolism exhibit the poorest prognoses and greater resistance to doxorubicin and docetaxel.

**Abbreviations**
AUC: area under the curve
CDF: cumulative distribution function
CNN: convolutional neural network
CNV: copy number variation
ER: oestrogen receptor
GSVA: gene set variation analysis
HER‐2: human epidermal growth factor‐2
KEGG: Kyoto Encyclopedia of Genes and Genomes
KS: Kolmogorov–Smirnov
MLP: Multilayer Perceptron
MODIN: Multiomics Drug‐cell Interaction Network
MSE: mean square error
PCA: principal component analysis
PR: progesterone receptor
SMILES: simplified molecular‐input line‐entry system
TNBC: triple‐negative breast cancer

## 1 Introduction

Triple‐negative breast cancer (TNBC) is a subtype of breast cancer that is characterised by a lack of oestrogen receptor (ER), progesterone receptor (PR), and human epidermal growth factor receptor 2 (HER2) expression [1]. Unlike other subtypes of breast cancer, TNBC does not respond to targeted therapies such as hormone therapy or HER2‐targeted therapy. Thus, TNBC patients tend to have a greater rate of relapse and a worse prognosis than those with other subtypes of breast cancer [2, 3]. In this context, predicting TNBC patient sensitivity to different chemotherapy drugs is essential for improving local control rates and prognosis.

Currently, the most widely used chemical information representation methods include SMILES (Simplified Molecular Input Line Entry System) and InChI (International Chemical Identifier) [4, 5]. SMILES uses simple ASCII character sequences to represent the topology of molecules, including atoms, bonds, rings, and branches. In contrast, InChI employs a hierarchical encoding system to provide detailed descriptions of a molecule's formula, connectivity, hydrogen positions, and stereochemistry. Notably, the increasing availability of advanced artificial intelligence technologies has enabled the application of deep learning models to predict drug sensitivity based on SMILES or InChI [6, 7]. For example, Graph Neural Networks (GNNs) have been particularly effective in modelling molecular structures, while transfer learning has addressed data scarcity issues. Attention mechanisms further enhance the identification of critical features influencing drug response, and techniques like SHAP improve model interpretability [8]. For instance, Li Peng et al. performed the MPCLCDA model, which effectively predicts the potential associations between circRNA and diseases by utilising automatically selected meta‐paths and contrastive learning [9].

In the realm of drug response prediction, particularly for triple‐negative breast cancer (TNBC), some computational methods have been developed to address the challenge of predicting how patients will respond to specific treatments. For example, The HIWCF (a hybrid interpolation weighted collaborative filtering) algorithm improves the performance of drug response prediction models by integrating gene expression profiles and drug response data using a weighted similarity matrix [10]. Specifically, HIWCF first calculates the gene expression similarity and drug response similarity between cell lines, and then combines these two similarity matrices to construct a comprehensive similarity matrix. Subsequently, it employs value weighting to adjust and balance the influence of different similarity data, thereby suppressing noise and enhancing prediction accuracy and model robustness. Furthermore, Hui Liu et al. performed NCFGER (a neighbour‐based collaborative filtering with global effect removal) to improve predictability by removing global effects and shrinking similarity scores, thus focusing on the most relevant neighbours for prediction [11]. And SNRMPACDC AntiCancer Drug Combination prediction (SNRMPACDC) processes drug features through a Siamese network and random matrix projection, combining these with cell line features to predict drug combinations' effectiveness [12]. Noticeably, Xue Piao et al. proposed an innovative weighted graph regularised matrix factorization (WGRMF) approach to predict the sensitivity responses of cancer cell lines to anticancer drugs. Their methodology introduced a p‐nearest neighbour graph sparsification technique to denoise the drug chemical structure similarity matrix (based on the Jaccard coefficient) and the cell line gene expression similarity matrix (based on the Pearson correlation coefficient), effectively retaining critical neighbourhood information. By constructing a weighted graph regularisation term, they integrated the sparsified similarity matrices into a matrix factorization framework, combined with Tikhonov regularisation. This hybrid strategy enabled the model to capture latent low‐dimensional features of drugs and cell lines while enhancing robustness against noise and data sparsity [13].

However, the use of similarity scoring in predicting drug IC50 values in a previous study presents several notable limitations. Primarily, the differences in modalities can lead to misalignment in the semantic representation space, which hampers the accurate reflection of the true relationship between drugs and cell line responses. Additionally, such methods often exhibit poor generalisation capabilities, resulting in sub‐optimal performance when applied to novel drugs or cell lines, thereby struggling to adapt to the complexities of biological environments. Furthermore, similarity scoring is sensitive to noise in the data, which can be influenced by experimental errors, adversely affecting the accuracy of predictions.

Distinct from these methods, which primarily rely on similarity scoring to model drug response relationships, our proposed Multiomics‐Driven Drug‐Cell Interaction Network (MODIN) introduces a novel framework that leverages cross‐modal attention mechanisms to seamlessly combine features from both cell lines and drug modalities. In this study, we addressed these issues by analysing the chemical and physical characteristics of 621 drugs from the PubChem dataset. We transformed the Simplified Molecular Input Line Entry System (SMILES) of these drugs into 2048‐dimensional fingerprints via the Rdkit library. Moreover, we standardised the different physicochemical properties and combined them to construct the input data for drug modelling. To incorporate the sequencing data of patients more effectively, we merged multiple types of omics data, including mRNA expression, copy number variation (CNV), and DNA methylation data, at the pathway level based on 186 distinct pathways found in KEGG datasets. The experimental results show evidence that our method achieves state‐of‐the‐art performance in contrast with other methods.

## 2 Methods

### 2.1 TNBC Patients and Cell Data Accession

Clinical data and RNA‐Seq profiles of triple‐negative breast cancer (TNBC) patients were obtained from The Cancer Genome Atlas (TCGA) datasets. We selected patients meeting the following criteria: (1) histologically confirmed TNBC status (ER‐negative, PR‐negative, HER2‐negative per ASCO/CAP guidelines), (2) availability of complete clinical annotations, and (3) paired RNA‐Seq data. This yielded 120 eligible TNBC cases for subsequent Gene Set Variation Analysis (GSVA) analysis.

We further curated TNBC cell lines from the Genomics of Drug Sensitivity in Cancer (GDSC) database based on the following criteria: (1) availability of dose–response curves (IC50 values) for 621 compounds, (2) RMA‐normalised mRNA expression profiles, and (3) complete multi‐omics datasets including Illumina Infinium MethylationEPIC arrays and Affymetrix SNP 6.0‐based copy number variation (CNV) data. 38 cell lines finally met these requirements and were used to train the Multi‐Omics Driven Interaction Network (MODIN). The validated model was then applied to 70 TCGA TNBC patients possessing matched DNA methylation (Infinium HumanMethylation450), RNA‐Seq (Illumina HiSeq), and CNV (Affymetrix SNP 6.0) profiles for drug sensitivity prediction.

The full DNA methylation profile, RMA‐normalised mRNA expression and CNV data for the TNBC cells and patients, denoted as D, M, and $C\in {\mathbb{R}}^{N\times K}$, respectively, were acquired from CCLE datasets (https://depmap.org/portal/download/custom/), where *N* is the number of cells and *K* denotes the number of genes in the list.

To integrate copy number variation (CNV), mRNA expression, and DNA methylation data into biologically meaningful representations, we performed pathway‐centric feature engineering through the following steps. First, 52 metabolic pathways were curated from the MSigDB Database (https://www.gsea‐msigdb.org/gsea/msigdb/index.jsp), Genes lacking concurrent measurements across all three omics platforms were excluded, retaining 12,751 shared genes for downstream analysis. Following pathway indexing, data quality control, and preprocessing, the mRNA, CNV, and methylation data were formatted into the matrix in ${\mathbb{R}}^{N\times d_{0}}$ shape, DNA methylation profile ($D_{\mathrm{pi}}\in {\mathbb{R}}^{N\times d_{0}}$), $\text{mRNA}\;\left(M_{\mathrm{pi}}\in {\mathbb{R}}^{N\times d_{0}}\right)$ and CNV ($C_{\mathrm{pi}}\in {\mathbb{R}}^{N\times d_{0}}$). Where n denotes cell samples and $d_{0}$ is the number of genes in the pathway. Then, we stack these three matrices and extend them by one dimension, result in a 3‐D shape features with ${\mathbb{R}}^{3\times N\times d_{0}}$.

To ensure effective indexing of each pathway, for genes with missing values exceeding 50% within a pathway, we employed mean imputation to fill the missing values with the mean expression of present entries. After processing the structured data, we applied p‐norm normalisation separately to eliminate distribution differences and the influence of noise.

We next obtained the IC50 values of *M* compounds for *N* TNBC cells as our regression labels $Y=\left\{\left(x_{i},z_{i},y_{i}\right),i=1,2,\cdots ,L\right\}$ (where x denotes the cell and z denotes the drug) from the GDSC1 and GDSC2 datasets (https://www.cancerrxgene.org/). The original IC50 values were log‐transformed for normalisation and are termed LN_IC50. After data cleaning (removing duplicates and missing items), *N* = 38, *M* = 621, and *L* = 20,411.

The molecular chemical formulas of the 621 compounds were downloaded from the PubChem datasets. We encoded the compounds' initial embedding (in the shape of $Z\in {\mathbb{R}}^{M\times d}$) from their SMILES by the open Rdkit Chem library.

We treated cell knowledge ($X\in {\mathbb{R}}^{3\times N\times K}$) and drug knowledge ($Z\in {\mathbb{R}}^{M\times d}$) as explanatory variables and LN_IC50 estimates as the decision variables via a regression framework. The final matrix consisted of 38 cell lines, each with a genetic signature of DNA methylation, mRNA, and CNV, as well as 621 compounds. A total of 20,411 instances of “genetic signature + drug information” were included in the final inputs to train the Multiomics Drug‐cell Interaction Network model. To train and evaluate our proposed model, we split the 20,411 labelled instances into training/test sets, as indicated by the 80%/20% protocol.

Five independent runs were conducted using distinct random seeds (42, 1234, 2023, 3407, 619) for stratified training‐test splits (8:2). And we report the average results in our experiments to ensure the reliability of our findings.

We implement our model using PyTorch 2.0.1, both training and testing are performed on CUDA 11.4. We utilise the Adam optimizer with an initial learning rate of 0.001, and the batch size is set to 32; the overall network layers are 6. The input and output dimensions depend on the form of the data, and we set the hidden layer dimension to 512 for representation learning. We set the training epochs to 1000. All experiments are conducted on one NVIDIA 3090Ti‐24G GPU device.

### 2.2 Overall Pipeline of MODIN

**FIGURE 1:**
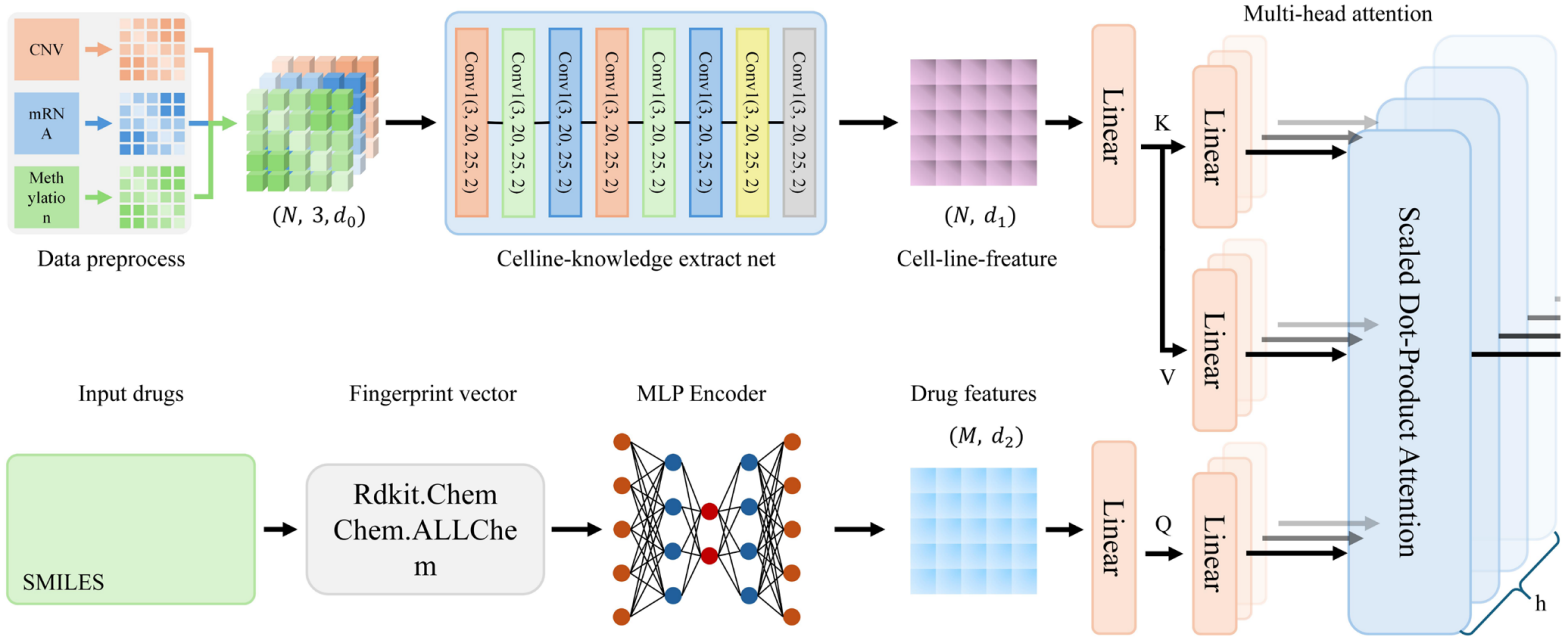
The overall pipeline of our model. The CNV, mRNA, and Methylation serve as cell‐line input, and the drug input is the smiles. Cell‐line knowledge extractor is used to extract the multi‐omics information, and Drug knowledge extractor outputs the drug features. Cross‐modal attentive interaction models the interaction between cells and drugs with an attention mechanism and maps the cell‐drug pairs into the same latent space. A prediction header is employed to predict the LN_IC50 values based on the latent space features.

The overall pipeline of our MODIN model is shown in Figure 1. The whole architecture of MODIN contains three parts: Cell‐line knowledge extractor, Drug knowledge extractor, and Cross‐modal attentive interaction. The model has two input modalities: gene expression data and drug information data. The gene expression data consists of three expression matrices, each corresponding to copy number variations (CNV), messenger RNA (mRNA), and methylation levels. The drug information data is represented as the molecular structure of the drug in SMILES format.

Our model ingests dual modalities: gene expression profiles and drug structures. Specifically, the gene expression data encompasses three distinct matrices representing CNV, mRNA, and methylation levels. Conversely, the drug information is encapsulated in the form of SMILES strings, which describe the molecular structure of the compounds.

The cell‐line knowledge extractor is pivotal for standardising and scaling the gene expression values, mitigating batch effects, and conducting feature selection to streamline dimensionality. This normalisation and feature reduction process is uniformly applied across the three gene expression matrices. The cell‐line knowledge extraction can be mathematically encapsulated as follows:

(1) $$H_{1}=f\left(X;\Theta_{1}\right)$$

(2) $$X=\text{concatenate}\;\left(\text{Norm}\left(C\right),\text{Norm}\left(M\right),\text{Norm}\;\left(D\right)\right)$$

where $H_{1}\in {\mathbb{R}}^{N\times d_{1}}$ denotes the cell‐line features, $\Theta_{1}$ is the convolutional neural network (CNN) parameter, $\text{Norm}\;\left(\cdot \right)$ is the p‐norm normalisation and $\text{concatenate}\;\left(\cdot ,\cdot ,\cdot \right)$ is the three‐element concatenation operation.

Subsequently, within the Drug Knowledge Extractor, the SMILES strings are meticulously transformed into molecular descriptors leveraging a sophisticated feature extraction algorithm such as RDKit. The transformation is mathematically represented as:

(3) $$H_{2}=f\left(Z;\Theta_{2}\right)$$

where $H_{2}\in {\mathbb{R}}^{M\times d_{2}}$ denotes the drug features and $\Theta_{2}$ is the parameter of the drug knowledge extractor.

**FIGURE 2:**
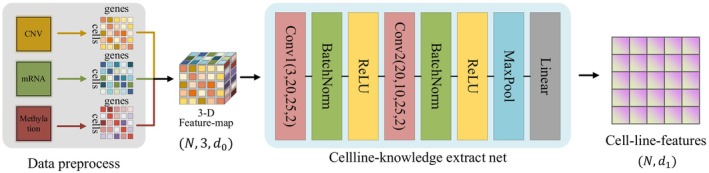
Cell‐line knowledge extractor. The multi‐omics cell data are preprocessed to construct a regular feature map. The extract net is a stacked CNN model and it contains the cnn layer, batch norm layer, the ReLU activation function, pooling layer, and the linear layer. The output is the cell‐line feature matrix.

Building upon the preprocessed data, the model then harnesses a cross‐modal attentive interaction module to scrutinise the interplay between gene expression, drug features, and the target LN_IC50 values. The predictive function is articulated as:

(4) $$Y=\mathrm{MHA}\left(H_{1},H_{2};\Theta_{3}\right)$$

where Y is the predicted LN_IC50 and $\Theta_{3}$ is the parameter of the multimodal hierarchical attention (MHA) mechanism, as delineated in Figure 2.

The subsequent sections will delve into the intricacies of these three neural modules, elucidating their contributions to the MODIN model's predictive prowess.

### 2.3 Cell‐Line Knowledge Extractor

The cell‐line knowledge extractor module is designed to extract meaningful features from three types of expression matrices: CNV, mRNA, and methylation. The input matrices have a size of $N\times d_{0}$, where N is the number of cells and $d_{0}$ is the number of genes in each matrix. The module first concatenated these matrices along the feature dimension, resulting in a 3‐D tensor size of $N\times 3\times d_{0}$. We reshaped the 3‐D tensor to $3\times N\times d_{0}$, which satisfies the input of the CNN model (3 is the number of channels, and $N\times d_{0}$ can be viewed as H×W according to the image data).

To eliminate the differences in data dimensions, we perform the row normalisation in each matrix individually. We used p‐norm normalisation, which calculates the p‐norm for each sample and then divides the norm for each element in the sample. This treatment resulted in the p‐norm (L1‐norm or L2‐norm) for each processed sample being equal to 1. The p‐norm can be calculated as follows:

(5) $${\left\|x\right\|}_{p}={\left({\left|x_{1}\right|}^{p}+{\left|x_{2}\right|}^{p}+\cdots +{\left|x_{d_{0}}\right|}^{p}\right)}^{\frac{1}{p}}$$

where x denotes the expression in one cell.

The normalised matrices are then concatenated to form the input tensor $X\in {\mathbb{R}}^{3\times N\times K}$, which is fed into a convolution neural network (CNN) for feature extraction. The CNN consists of two large‐kernel convolutional layers (the kernel size is 25×25) with batch normalisation and non‐linear activation function, followed by a max pooling layer and a fully connected layer. The architecture is represented in Figure 2, where Conv1 and Conv2 are the two convolutional layers, BatchNorm denotes batch normalisation, ReLU denotes the non‐linear activation function, MaxPool denotes the max pooling layer, and Linear denotes a fully connected layer. The output of the module was a feature map of size $N\times d_{1}$, where $d_{1}$ is the number of extracted features.

The CNN applied a set of learnable filters (kernels) to the input tensor X to capture the local patterns and interactions between the genes and methylation sites. Each filter had a receptive field (kernel size k=25) that defined the size of the local pattern that it can detect. The output feature map $H_{i}$ at position i was obtained by applying the filter $w_{i}$ to a local patch of the input tensor X centered at position i:

(6) $$H_{i}=f\left(w_{i}X_{\text{patch}}+b_{i}\right)$$

where f denotes the activation function, $w_{i}$ and $b_{i}$ are the learnable weights and bias of filter i, respectively, and $X_{\text{patch}}$ is a subtensor of X centered at position i of size k along each dimension.

### 2.4 Drug Knowledge Extractor

**FIGURE 3:**
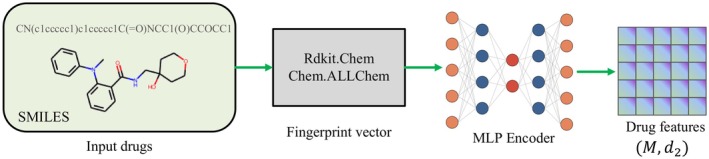
Drug knowledge extractor. Rdkit library is used to embed the input drugs into a fingerprint vector, which is a very sparse matrix. The MLP encoder is used to learn a drug feature matrix.

The Drug Knowledge Extractor Module was designed to extract meaningful features from drug molecules represented as SMILES strings. As shown in Figure 3, the module first calls the Rdkit. Chem library, which was used to obtain a 2048‐dimensional intermediate embedding of the drug molecule. Due to the sparsity of embedding, a multilayer perceptron (MLP) is used to extract the drug representation, which had a size of $M\times d_{2}$.

Formally, given a drug molecule represented as a SMILES string, the Rdkit. Chem library was used to generate a 2048‐dimensional intermediate embedding h of the drug molecule:

(7) $$H_{2}=f\left(\text{Rdkit}.\text{Chem}\left(\text{SMILES}\right)\right)$$

where f denotes a nonlinear activation function applied elementwise to the intermediate embedding.

**FIGURE 4:**
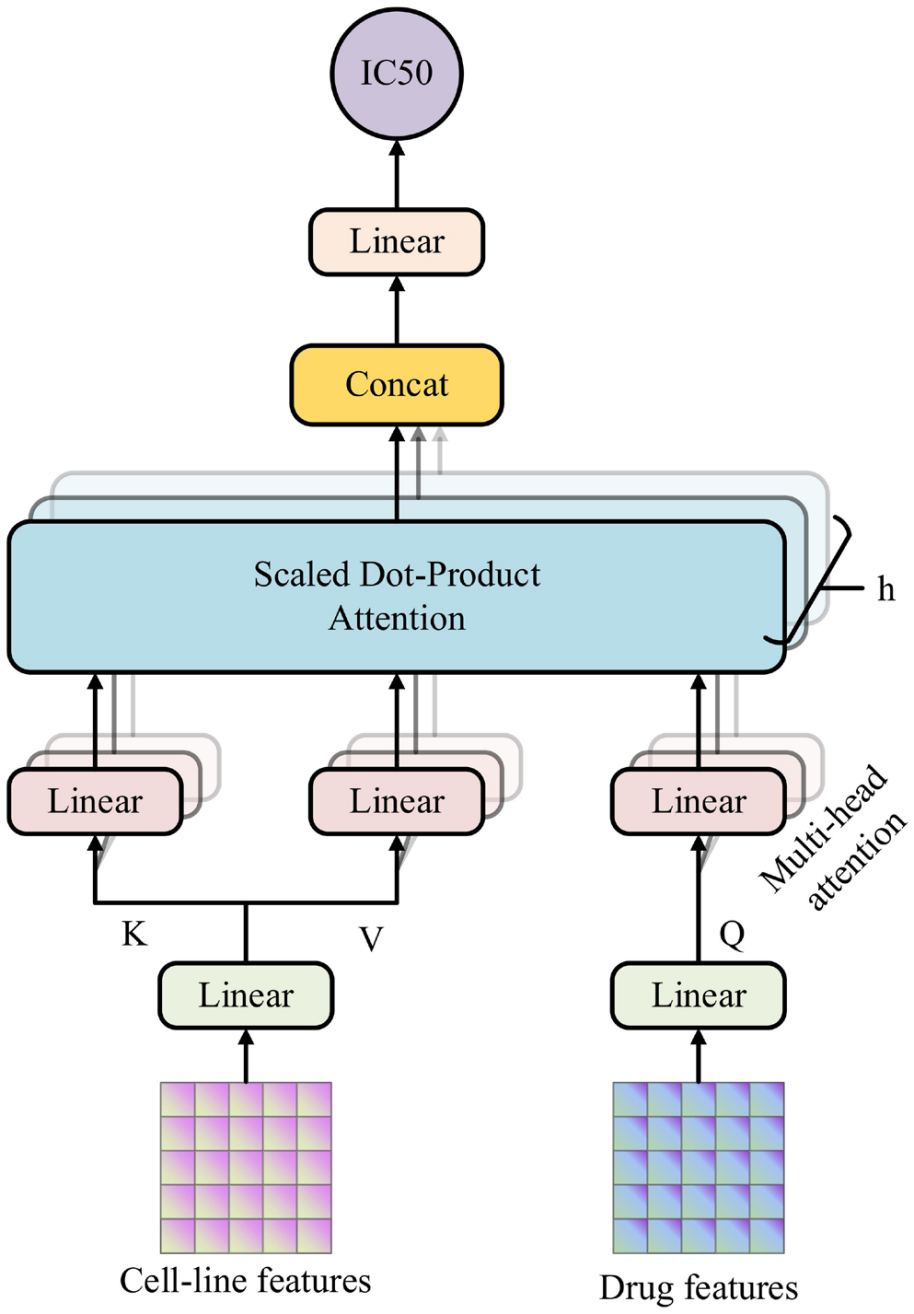
Cross‐modal attentive interaction follows the principle of a multi‐head attention mechanism. The Query (Q) and Key (K) are calculated from cell‐line features and the Value (V) is calculated from drug features. We concatenate the output of the h heads and predict the cell‐drug pair's IC50 value.

To obtain the drug representation, the intermediate embedding h was passed through an MLP, which consisted of L fully connected layers with nonlinear activation functions:

(8) $$H_{2}^{\prime }=\mathrm{MLP}\;\left(H_{2}\right)=f_{L}\left(f_{L-1}\left(\ldots f_{1}\left(h\right)\right)\right)$$

where $f_{l}$ denotes the activation function of layer l and ${H_{2}}^{\prime }$ is the output of the MLP, which represents the drug in a lower‐dimensional space. The size of the output vector was $M\times d_{2}$, where M is the number of drugs and $d_{2}$ is the number of features extracted by the MLP (Figure 4).

### 2.5 Cross‐Modal Attentive Interaction

This module adeptly leverages a multi‐head attention mechanism to assess the correlation between the two modalities, facilitating a deeper understanding of drug‐cell interactions. Cross‐modal attentive interaction begins with the transformation of cell‐line features to generate the Key and Value, while the Query vector is derived from the drug features (Figure 4).

In this framework, the cell‐line features ($H_{1}$) serve as the source for generating the Key (K) and value (V) vectors, while the drug features ($H_{2}$) provide the Query (Q) vector. Which are projected with linear networks:

(9) $$K=W_{k}\;H_{1}+b_{k},V=W_{v}\;H_{1}+b_{v},Q=W_{q}\;H_{2}+b_{q}.$$

where $\left\{W,b\right\}$ are the learnable parameters. These representations are then passed into a multi‐head attention mechanism, which computes the correlations between the drug and cell‐line features. For one head, it can be formulated as:

(10) $$A=\text{Attention}\;\left(Q,K,V\right)=\text{softmax}\;\left(\frac{Q\;K^{T}}{\sqrt{d_{k}}}\right)\;V$$

where $d_{k}$ is the dimensionality of the Key, and $\text{softmax}\;\left(\cdot \right)$ is the softmax function. The output of the multi‐head attention layer captures the cross‐modal interactions, with outputs from multiple heads being concatenated and passed through a final linear layer to predict the LN_IC50 values:

(11) $$Y_{\text{pred}}=W_{y}\;\left[A_{1};A_{2};\ldots ;A_{h}\right]+b_{y}$$

where $\left[A_{1};A_{2};\ldots ;A_{h}\right]$ denotes the concatenation of the outputs of h heads and $W_{y}$ and $b_{y}$ are learnable parameters of the linear layer.

This cross‐attention mechanism enables the MODIN model to effectively capture the nuanced interactions between the two modalities. By combining this module with a convolutional neural network (CNN) for feature extraction and a multi‐layer perceptron (MLP) for dimensionality reduction, MODIN achieves state‐of‐the‐art performance in predicting LN_IC50 values. The model is trained end‐to‐end using a loss function, such as Mean Squared Error (MSE), and optimised via backpropagation.

### 2.6 Model Evaluation Metrics

In this study, we used the MSE, root mean squared error (RMSE) and coefficient of determination (*R* ^2^) as the evaluation metrics to assess the performance of the model. The MSE is the average of the squared differences between the predicted values and the true values, and a lower MSE indicates better predictive ability. The *R* ^2^ indicates the proportion of variance in the data that is explained by the model, with a value closer to 1 indicating greater explanatory power.

The formulas for MSE and *R* ^2^ are as follows:

(12) $$\mathrm{MSE}=\frac{1}{N}\;\sum_{i=1}^{N}{\left(y_{i}-{ŷ}_{i}\right)}^{2}$$

(13) $$R^{2}=1-\frac{1}{N}\sum_{i=1}^{N}\frac{{\left(y_{i}-{ŷ}_{i}\right)}^{2}}{\sum_{i=1}^{N}{\left(y_{i}-ȳ\right)}^{2}}$$

The formulas for the RMSE are as follows:

(14) $$\text{RMSE}=\sqrt{\frac{1}{n}\sum_{i=1}^{N}{\left(y_{i}-{\hat{y}}_{i}\right)}^{2}}$$

where N is the number of cell‐drug pairs, $y_{i}$ is the true value of LN_IC50, ${ŷ}_{i}$ is the predicted value, and ȳ is the mean of the true values. We used the MSE as the training objective function and evaluated the performance of the model by computing the MSE and *R* ^2^ of the test set.

All tests were two‐sided with a level of significance set at *p* < 0.05.

### 2.7 Consensus Clustering

In this study, we applied Consensus Clustering as follows: (1) Resampling: The dataset was resampled 1000 times to create diverse subsets. (2) Clustering Algorithm: K‐means clustering was applied to each subset with *k* ranging from 2 to 10. (3) Consensus Matrix: The consensus matrix was computed for each *k* and analysed. (4) Evaluation Metrics: The CDF distribution and delta area plots were used to assess the stability and determine the optimal *k*.

As presented in Figure 1B, the distribution of the cumulative distribution function (CDF) for different numbers of clusters, *k*. Higher values indicate more robust clustering results for that particular *k*. The Delta area plot (right panel) displays the relative change in the area under the CDF curve between k and k − 1. Larger values suggest a significant improvement in clustering quality for *k* compared to *k* − 1. If the change in area between adjacent *k* values is minimal, it indicates that further increasing k does not significantly enhance clustering stability. The CDF distribution becomes more stable for *k* ≥ 4, while the improvement in the area under the CDF curve for *k* = 5 is less pronounced compared to *k* = 4, suggesting that the clustering solution with *k* = 4 should be considered.

### 2.8 Targeted Metabolomics

Targeted Metabolomics was performed by Guangzhou Genedenuo Biotechnology Co. Ltd. (https://www.omicshare.com/). The analysis was performed using a Waters Acquity UPLC system coupled with an AB SCIEX 5500 QQQ‐MS mass spectrometer. For chromatographic separation, we employed Acquity UPLC BEH C18 columns (1.7 μm, 2.1 mm × 100 mm) and Acquity UPLC HSS T3 columns (1.8 μm, 2.1 mm × 100 mm). Additionally, a Thermo Trace1300 gas chromatograph interfaced with an ISQ7000 mass spectrometer was used, equipped with a DB‐5 ms column.

The chromatographic conditions were optimised with a column temperature set at 35°C and a flow rate of 0.30 mL/min. The mobile phase was composed of water containing 10 mM ammonium formate and methanol. The mass spectrometric conditions were as follows: an ESI ion source, curtain gas at 35 arb, collision gas at 7 arb, an ion spray voltage of 4500 V, and an ion source temperature of 450°C.

### 2.9 Free Fatty Acid Uptake Assay

Free Fatty Acid Uptake Assay Kit (Fluorometric) (ab176768) provides a simple and sensitive method for the measurement of fatty acid uptake in cells containing fatty acid transporters. The kit uses a proprietary dodecanoic acid fluorescent fatty acid substrate. This fatty acid uptake assay kit can be performed on any fluorescence microplate reader with a bottom‐read mode at Ex/Em = 485/515 nm or FITC channel. Cells were plated at 50,000 cells/100 μL/well in a 96 well black wall/clear bottom poly‐D lysine plate for 5 h and then serum deprived for 1 h. Cells were treated without (control) or with insulin (150 nM) and incubated at 37°C, 5% CO_2_ incubator for 30 min. At the end of the incubation time, 100 μL of fatty acid mixture was added into the well and incubated for another 60 min; the fluorescence signal was measured with a plate reader using bottom read mode.

### 2.10 Statistical Analyses

Fisher's exact test was used to analyse the clinicopathological characteristics among breast cancer patients in the MS_1 to MS_4 groups, with a *p*‐value < 0.05 considered statistically significant for intergroup differences. The Kolmogorov–Smirnov (KS) test was utilised to examine whether the IC50 values of the four patient groups were derived from a common sample distribution. The log‐rank test was used to assess the statistical significance of differences in survival among the four patient groups. A *p* < 0.05 was used to define statistical significance.

## 3 Results

### 3.1 Metabolism‐Based Subtyping of TNBC

**FIGURE 5:**
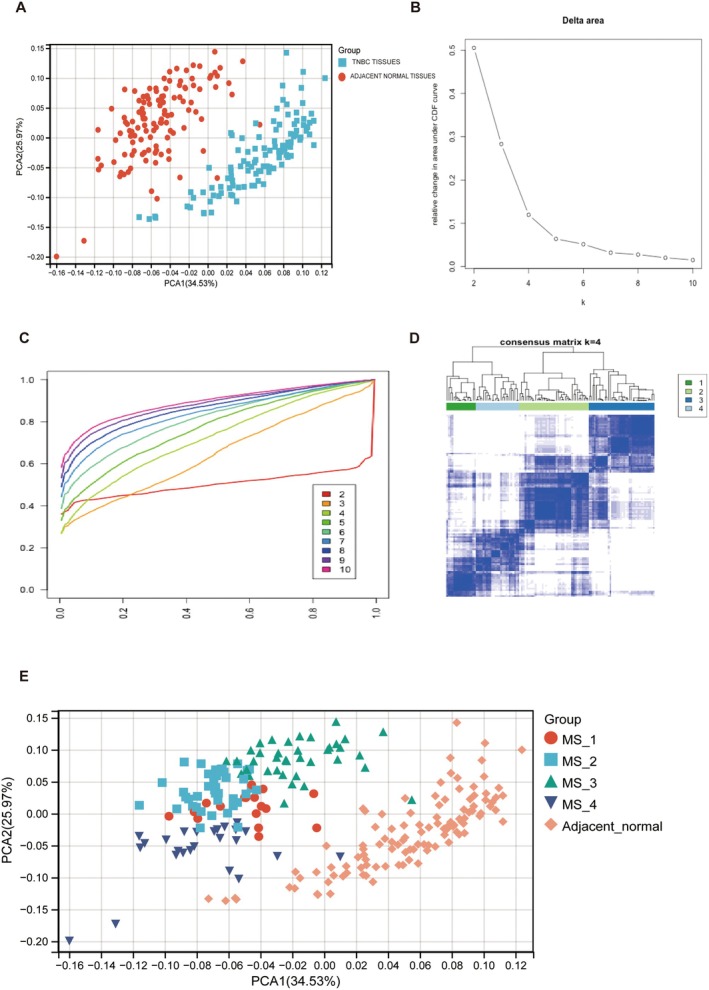
(A) The PCA plot of enrichment scores for 52 metabolic pathways in Triple‐Negative Breast Cancer (TNBC) tumour samples and adjacent normal tissues. (B) Proportion changed in the area under the CDF (Cumulative Distribution Function) curves. (C) Empirical CDFs corresponding to the entries of consensus matrices M(K) for *k* = 2, 3, 4, …10. (D) The heatmap of the Consensus Matrix for Consensus Clustering at *k* = 4. Each cell quantifies the degree to which data points exhibit consistent assignments to the same cluster, and this consistency is visually encoded using a colour scale that transitions from white (indicating low consensus) to blue (indicating high consensus) to represent the consensus values. (E) The PCA plot of enrichment scores for 52 metabolic pathways in four metabolic subtypes (MS_1, MS_2, MS_3, and MS_4) within Triple‐Negative Breast Cancer (TNBC) tumour samples and adjacent normal tissues.

A total of 12,751 genes associated with the 52 metabolic pathways were obtained from KEGG datasets. GSVA was used to determine the enrichment scores of 52 metabolic pathways in 120 TNBC tissues and 110 adjacent normal tissues in the TCGA datasets. Principal component analysis (PCA) was conducted based on the enrichment of 52 metabolic pathways in 120 TNBC tumour tissues and 110 TNBC adjacent tissues (Figure 5A).

The study revealed distinct metabolic alterations in TNBC compared to normal samples. To further investigate these metabolic differences, we employed Consensus Cluster Plus, an unsupervised class discovery tool that utilises the enrichment scores of 52 metabolic pathways among 120 TNBC patients. The Consensus Cluster Plus algorithm generated consensus matrices for K values ranging from 1 to 10. Subsequently, consensus clustering identified four distinct metabolic subtypes determined by the point where the consensus distribution curve began to plateau (*K* = 4). We then calculated the cumulative distribution using a histogram with 100 bins (Figure 5B,C). Additionally, we computed the relative change in the area under the curve (AUC) of the cumulative distribution function (CDF) between *K* and *K*−1. Points exhibiting insignificant increases were selected, with the optimal *K* value determined to be 4 (Figure 5D).

### 3.2 Patients With Different Subtypes Have Distinct Metabolic Features

**FIGURE 6:**
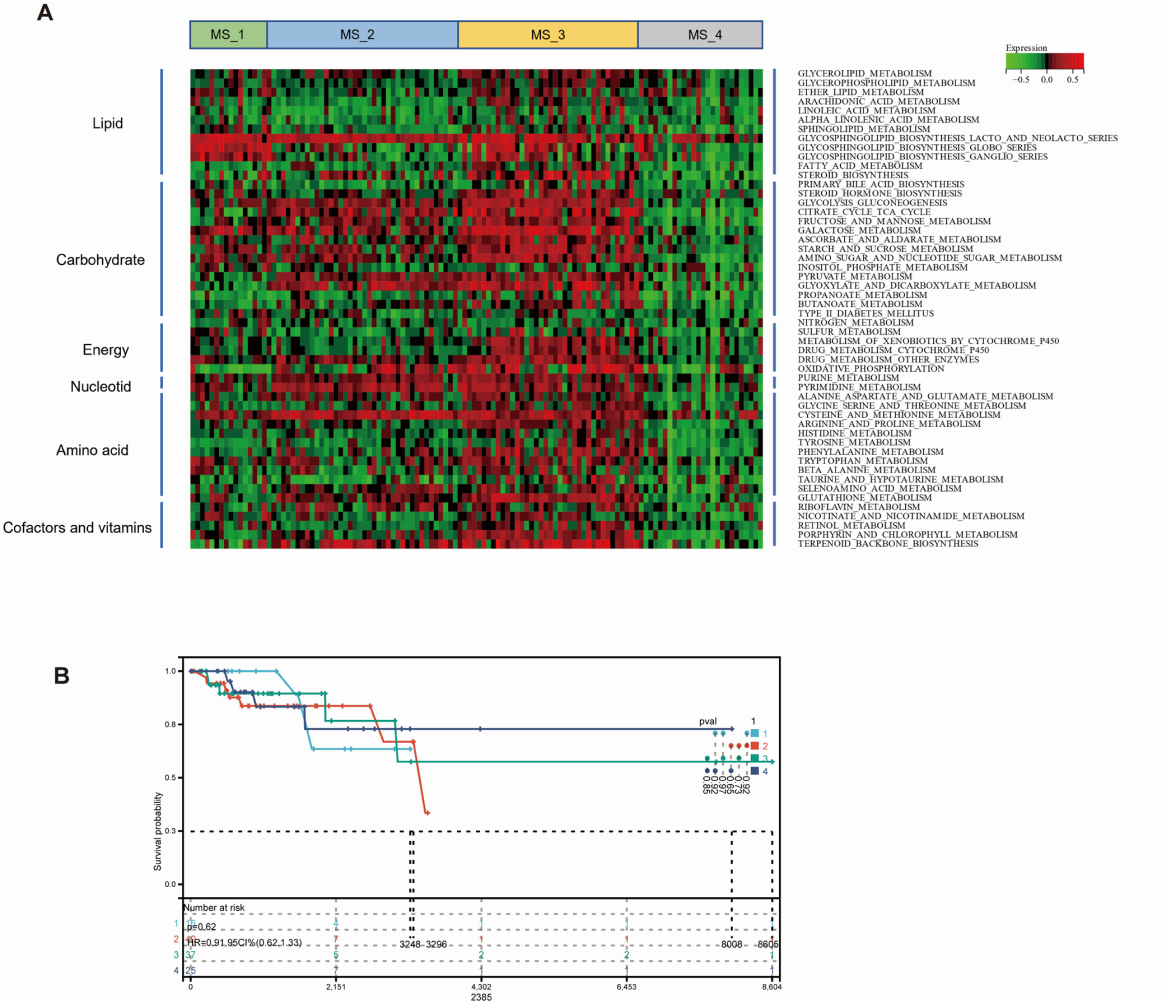
(A) Heatmap of GSVA scores for 52 metabolic pathways in 4 metabolic TNBC cohorts (MS_1, MS_2, MS_3 and MS_4). (B) Kaplan–Meier curves of overall survival among the four metabolic subtypes (MS_1, MS_2, MS_3, and MS_4) of Triple‐Negative Breast Cancer (TNBC) cohort.

A heatmap of the metabolic enrichment scores of patients from the 4 subtypes is depicted in Figure 6A. The MS_1 patients exhibited notable activation of glycosphingolipid biosynthesis, which played a crucial role in cellular communication and recognition. Additionally, moderate activation of carbohydrate metabolism and the inhibition of nucleotide metabolism were also observed in MS_1 patients. MS_2 patients displayed elevated activation of carbohydrate and nucleotide metabolism but suppressed lipid metabolism. This metabolic reprogramming indicated an adaptive strategy adopted by tumours to cope with fluctuations in the microenvironment and sustain rapid proliferation. MS_3 patients were characterised by significant activation of all types of metabolism, including lipid, carbohydrate, amino acid, and nucleotide metabolism. In contrast, MS_4 patients demonstrated suppressed metabolic activity across all types of metabolism. This may indicate reduced metabolic demands of tumour cells in MS_4 patients or limitations in metabolic capacity, possibly due to the influence of tumour suppressor factors or inherent defects in metabolic pathways.

Table 1 presented the clinical characteristics of the 120 TNBC patients. MS_3 patients were older than those in the other groups and had higher N stages. Among the four metabolic subtypes, MS_4 patients with suppressed metabolic activity exhibited the longest mean survival time (1655.56 ± 1717.78 months), whereas MS_2 patients with suppressed lipid metabolism but increased carbohydrate activation showed the shortest mean survival time (1170.75 ± 1029.40 months). However, the log‐rank test did not reveal any significant differences among the four groups of patients (Figure 6B).

**TABLE 1:** The clinical characteristic of 120 TNBC patients.

	MS_1	MS_2	MS_3	MS_4	*p*
Total	17	40	38	25	
T stage					
T1	8	6	5	5	0.06
T2	32	22	17	80	
T3	1	7	3	11	
T4	1	2	0	3	
TX	0	0	1	0	
N stage					
N0	12	28	2	1	0.27
N1	5	9	5	8	
N2	0	2	4	2	
N3	0	1	5	1	
M stage					
M0	17	38	34	24	0.7
M1/MX	0	2	4	1	
Event					
Alive	14	33	33	21	0.95
Dead	3	7	5	4	
Mean ± SD (months)	1170.75 ± 1029.40	1330.87 ± 1858.08	1655.56 ± 1717.78	1378.78 ± 1469.45	

### 3.3 The Multiomics Drug‐Cell Interaction Network (MODIN) Was Used to Predict Drug Response in TNBC Patients

**FIGURE 7:**
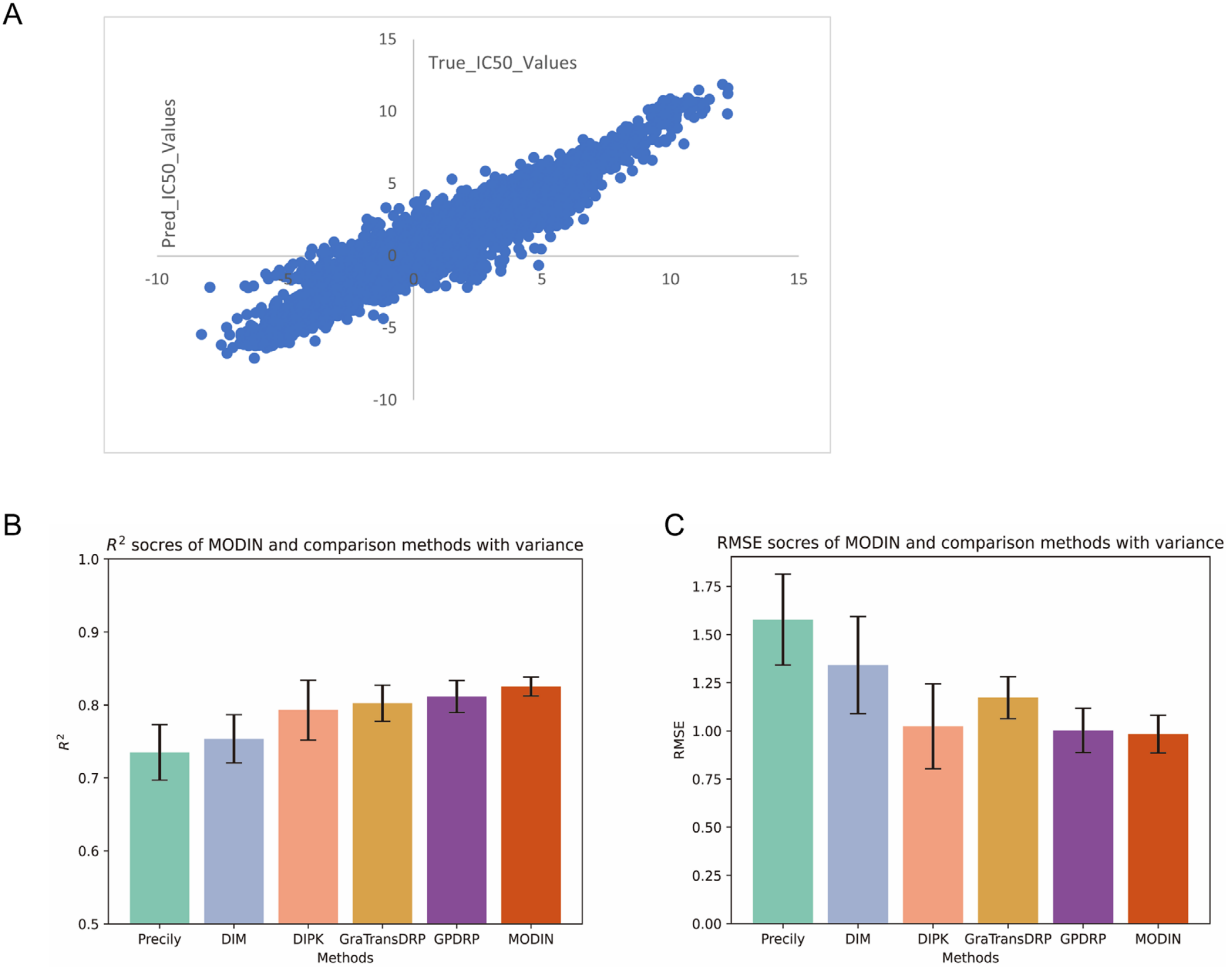
(A) The scatter plots of grand‐truth LN_IC50 values and MODIN predicted LN_IC50 values for 621 drugs (The X axis represented the grand‐truth LN_IC50 values and the Y axis represented the predicted LN_IC50 values. Pearson correlation score = 0.96, *p* values < 0.001***). (B, C) The *R* ^2^ and RMSE scores performance of MODIN with 5 state‐of‐the‐art methods (Precily, DIM, DIPK, GraTransDRP, GPDRP).

To determine the sensitivity of TNBC patients with different metabolic subtypes to chemotherapy drugs, we developed a deep learning framework, the MODIN, to accurately predict molecular targets. Molecular structure and chemical information were encoded by SMILES. MODIN embeds the multiomics gene information (mRNA expression, copy number variation and DNA methylation) of TNBC cells and drug SMILES information into a latent space and employs a multi‐head attention‐based interaction module to predict the LN_IC50 values of 621 drugs across 38 TNBC cell lines (a detailed description of MODIN's overall pipeline can be found in Method 3—the overall pipeline of MODIN). The Pearson's correlation coefficient between the LN_IC50 values predicted by MODIN and actual LN_IC50 values was 0.96 (*p* values < 0.001***, Figure 7A). In the regression analysis, the MODIN model demonstrated an *R* ^2^ value of 0.8254, an MSE value of 0.9672, and an RMSE value of 0.9835.

### 3.4 Benchmark Evaluation of MODIN

To determine which type of omics provided the most important and informative data for the model, we conducted MODIN modelling of data generated by combining data from two types of omics. Specifically, we combined mRNA expression data with CNV data, CNV data with DNA methylation data, and mRNA data with DNA methylation data. The *R* ^2^ values for these combinations were 0.8151, 0.8026, and 0.8129, respectively. The combination of CNV and DNA methylation data resulted in significantly worse performance. Furthermore, MODIN analysis of the single omics type resulted in *R* ^2^ values of 0.8085, 0.8059, and 0.8123 for CNV, mRNA, and methylation, respectively (Table 2). The model exhibited superior predictive performance when integrating all omics data; thus, the integration of all omic data led to the most accurate predictions. We next conducted the drug response for TNBC cell lines experiments in comparison with 5 state‐of‐the‐art methods, including Precily [14], DIM [15], DIPK [15], GraTransDRP [16], GPDRP [17]. The results of R2 and RMSE are reported in Table 3 and Figure 7B,C. As shown in the results, our method significantly outperforms the five sota baselines; compared to the well‐known Precily model, MODIN has a relative improvement of 12.31% in *R* ^2^ evaluation metric while reducing the RMSE error to 0.5964.

**TABLE 2:** The performance comparison of different multi‐omics combinations for cell‐line input.

Multi‐omics	MSE ↓	RMSE ↓	*R* ^2^ ↑
CNV only	1.2245	1.1066	0.8085
mRNA only	1.2408	1.1139	0.8059
Methylation only	1.2006	1.0957	0.8123
CNV + mRNA	1.1821	1.0872	0.8151
CNV + Methylation	1.2623	1.1235	0.8026
mRNA+Methylation	1.1964	1.0938	0.8129
CNV + mRNA+Methylation	0.9672	0.9835	0.8254

**TABLE 3:** The performance comparison of MODIN with 5 state‐of‐the‐art methods.

Methods	*R* ^2^	RMSE
Precily	0.7349 ± 0.038	1.5776 ± 0.236
DIM	0.7653 ± 0.041	1.2518 ± 0.221
DIPK	0.8023 ± 0.025	1.0237 ± 0.109
GraTransDRP	0.7931 ± 0.033	1.1725 ± 0.252
GPDRP	0.8116 ± 0.022	1.0028 ± 0.115
MODIN (Ours)	0.8254 ± 0.013	0.9835 ± 0.098

### 3.5 Training Convergence

**FIGURE 8:**
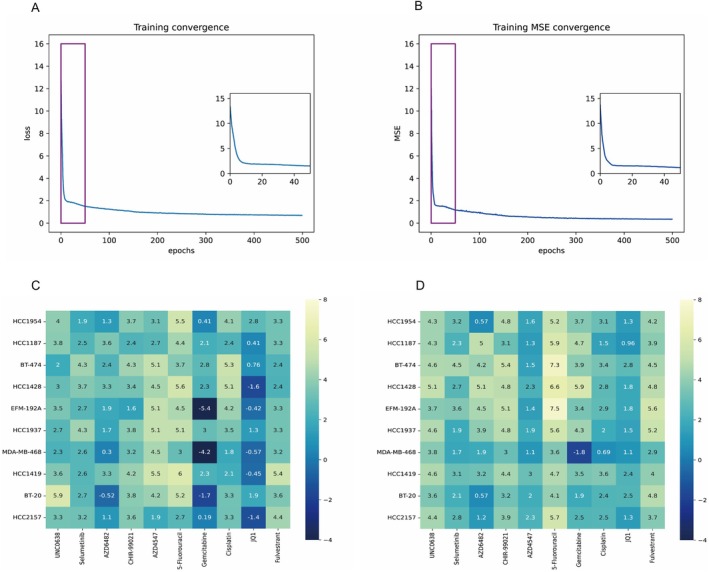
(A, B) The convergence behaviour of MODIN training. the training loss decreases process (A) and the test MSE convergence (B). (C, D) Heatmap of grand‐truth LN_IC50 values (C) and MODIN predicted LN_IC50 values (D).

We next calculated the test dataset's MSE and *R* ^2^ at every training epoch and plotted their curves. As shown in Figure 8A,B, as training progressed, the MSE decreased, and the *R* ^2^ value substantially increased, indicating that our model can converge without any perturbation and that the predictive ability can be improved by MODIN training. A heatmap of the predicted LN_IC50 values in relation to the true LN_IC50 values is presented in Figure 8C,D. The left side of the heatmap represents the true LN_IC50 values, while the right side represents the predicted LN_IC50 values. The drugs are listed on the horizontal axis, and the cells are listed on the vertical axis. Comparison of the true and predicted values showed that the model had good performance in predicting the drug sensitivity of different types of cells.

### 3.6 Patients With Different Metabolic Subtypes Showed Distinct Sensitivity to Various Drugs

**FIGURE 9:**
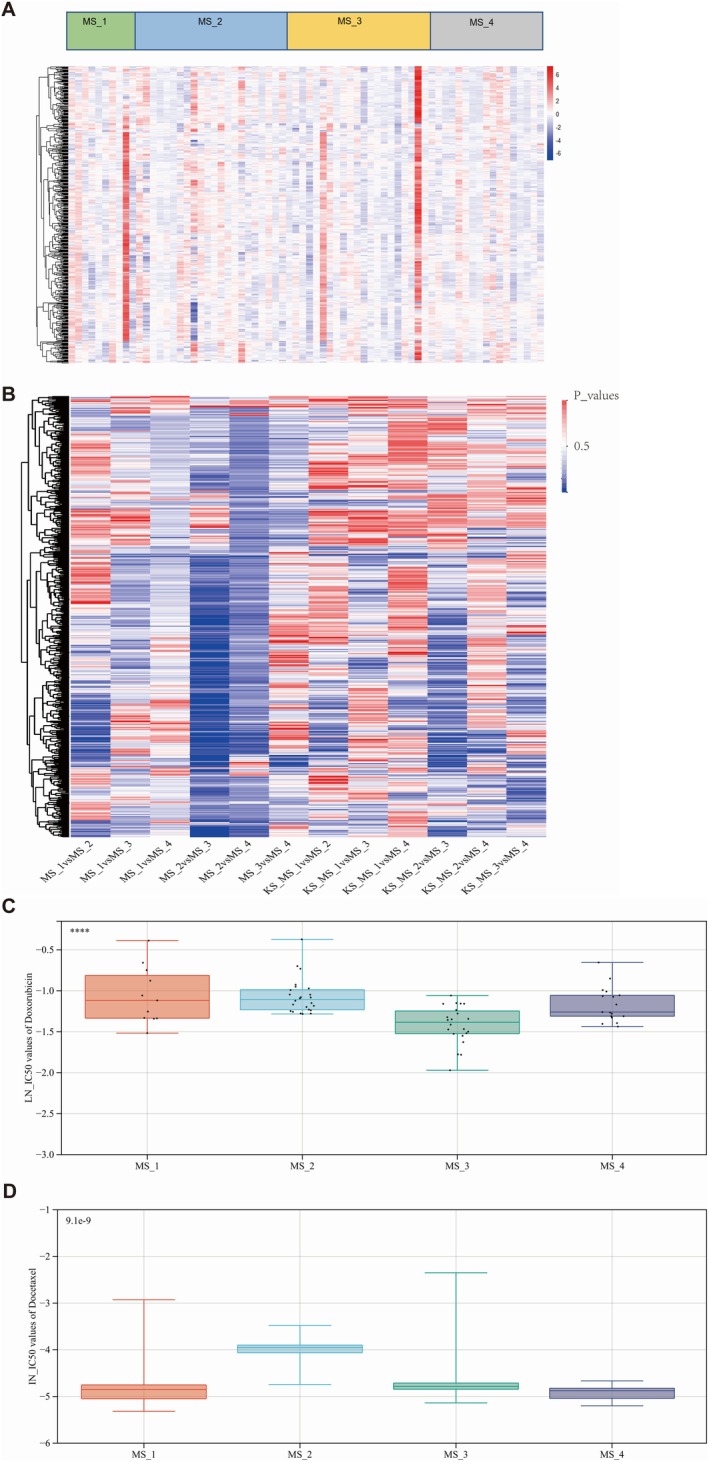
(A) The heatmap presentation of LN_IC50 values of the 621 drugs among 70 TNBC patients. (B) The *p*‐values for the Kolmogorov–Smirnov (KS) tests and independent *t*‐tests assessing the differences in predicted IC50 values of 621 chemotherapy drugs between the MS_1 and MS_2, MS_1 and MS_3, MS_1 and MS_4, MS_2 and MS_3, MS_2 and MS_4 groups in Triple‐Negative Breast Cancer (TNBC) patients. (C) The LN_IC50 values of Doxorubicin among MS_1, MS_2, MS_3, and MS_4 patients' cohorts. (D) The LN_IC50 values of Docetaxel among MS_1, MS_2, MS_3, and MS_4 patients' cohorts.

To assess the sensitivity of patients with four distinct subtypes of breast cancer to various chemotherapy drugs, we obtained mRNA‐seq, DNA methylation, and CNV data for TNBC patients from TCGA datasets. Ultimately, a cohort of 70 TNBC patients with comprehensive multi‐omics information, which included 15,667 genes, was included in the analysis. The MODIN model was used to predict the IC50 values of the 70 TNBC patients for a set of 621 drugs. A heatmap of the LN_IC50 values of the 621 drugs among 70 patients is presented in Figure 5A. The most sensitive and most resistant drugs in all patients are listed in Tables 4 and 5, respectively. Based on the MODIN, the 5 drugs that TNBC patients had the most resistance to included ascorbate (vitamin C) (IN_IC50: 10.64), N‐acetyl cysteine (IN_IC50: 10.24898), glutathione (IN_IC50: 9.16), alpha‐lipoic acid (IN_IC50: 7.67), and phenformin (IN_IC50: 7.18). Furthermore, TNBC patients were resistant to cetuximab (IN_IC50: 5.86), oxaliplatin (IN_IC50: 5.27), and 5‐fluorouracil (IN_IC50: 5.17). The 5 drugs that TNBC patients were most sensitive to included romidepsin (IN_IC50: −5.44196), bortezomib (IN_IC50: −4.85979), docetaxel (IN_IC50: −4.85679), thapsigargin (IN_IC50: −4.67304), and dactinomycin (IN_IC50: −4.11025). KS tests and independent t tests were used to compare drug sensitivity among different metabolic groups. The P values of the KS test and t test when comparing each of the 2 groups are presented in a heatmap. We found that there was the greatest disparity between MS_2 patients and MS_3 patients (Figure 9B). We compared the IC50 values of therapeutic agents in the classic chemotherapy regimen of TNBC between patients in the MS_2 and MS_3 cohorts. The MS_3 cohort exhibited greater sensitivity to chemotherapy regimens, including those with doxorubicin and docetaxel, than the MS_2 cohort (Figure 9C,D).

**TABLE 4:** The top 20 most resistant drugs for TNBC patients based on MODIN prediction.

Drug	IN_IC50	Targets
ascorbate (vitamin C)	10.64463	SREBF1
N‐acetyl cysteine	10.24898	SREBF2
Glutathione	9.161219	A1BG
Alpha‐lipoic acid	7.671816	ABCA1
Phenformin	7.180038	Glutathione S‐transferase A3
AICA Ribonucleotide	7.056355	Glutathione S‐transferase Mu 1
DMOG	7.010539	SAE1
Cetuximab	5.869792	NFE2L2
Nelarabine	5.767867	PRKAA1
BEN	5.518214	REG3G
Temozolomide	5.481825	Bifunctional purine biosynthesis protein ATIC
Selisistat	5.400285	CASP3
eEF2K Inhibitor, A‐484954	5.306488	KDM4D
Oxaliplatin	5.27872	HIF1A
GSK2830371	5.253601	DNA damage
Zibotentan	5.224598	Adenosine deaminase
5‐Fluorouracil	5.173415	Deoxycytidine kinase
ETP‐45835	5.153521	MSH6 NEK1
LMB_AB1	5.138685	
EPZ5676	5.126679	NAD‐dependent protein deacetylase sirtuin‐1 PDX1 Protein phosphatase 1D Endothelin‐1 receptor ECE1 CCND1 FOXO1 Histone‐lysine N‐methyltransferase, H3 lysine‐79 specific

**TABLE 5:** The top 20 most sensitive drugs for TNBC patients based on MODIN prediction.

Drug	IN_IC50	Targets
Romidepsin	−5.44196	HDAC6
Bortezomib	−4.85979	ERBB2
Docetaxel	−4.85679	PSMA7
Thapsigargin	−4.67304	PSMB3
Dactinomycin	−4.11025	AKR1C3
Vinblastine	−3.94228	EGFR
Vinorelbine	−3.86849	MBNL2
AZD4877	−3.68224	MCCC2
Sepantronium bromide	−3.41464	ESR1
Omipalisib	−3.04912	NR3C1
SN‐38	−3.00323	TUBB
Bryostatin 1	−2.89491	CGA
Eg5_9814	−2.8854	TUBA1C
Paclitaxel	−2.85461	TUBA1A
Paclitaxel	−2.85405	Kinesin‐like protein KIF11
SN‐38	−2.80001	Kinesin‐like protein KIF11
Dacinostat	−2.74644	ELAVL1
Panobinostat	−2.5632	MAP1LC3B
NSC319726	−2.48081	PIK3R2
Staurosporine	−2.4412	PIK3R5 PRKCA PRKCE BIRC5 SOX10 Histone deacetylase 5 Histone deacetylase 7 Histone deacetylase 4 Histone deacetylase 7 CSF2 IL2RA

**FIGURE 10:**
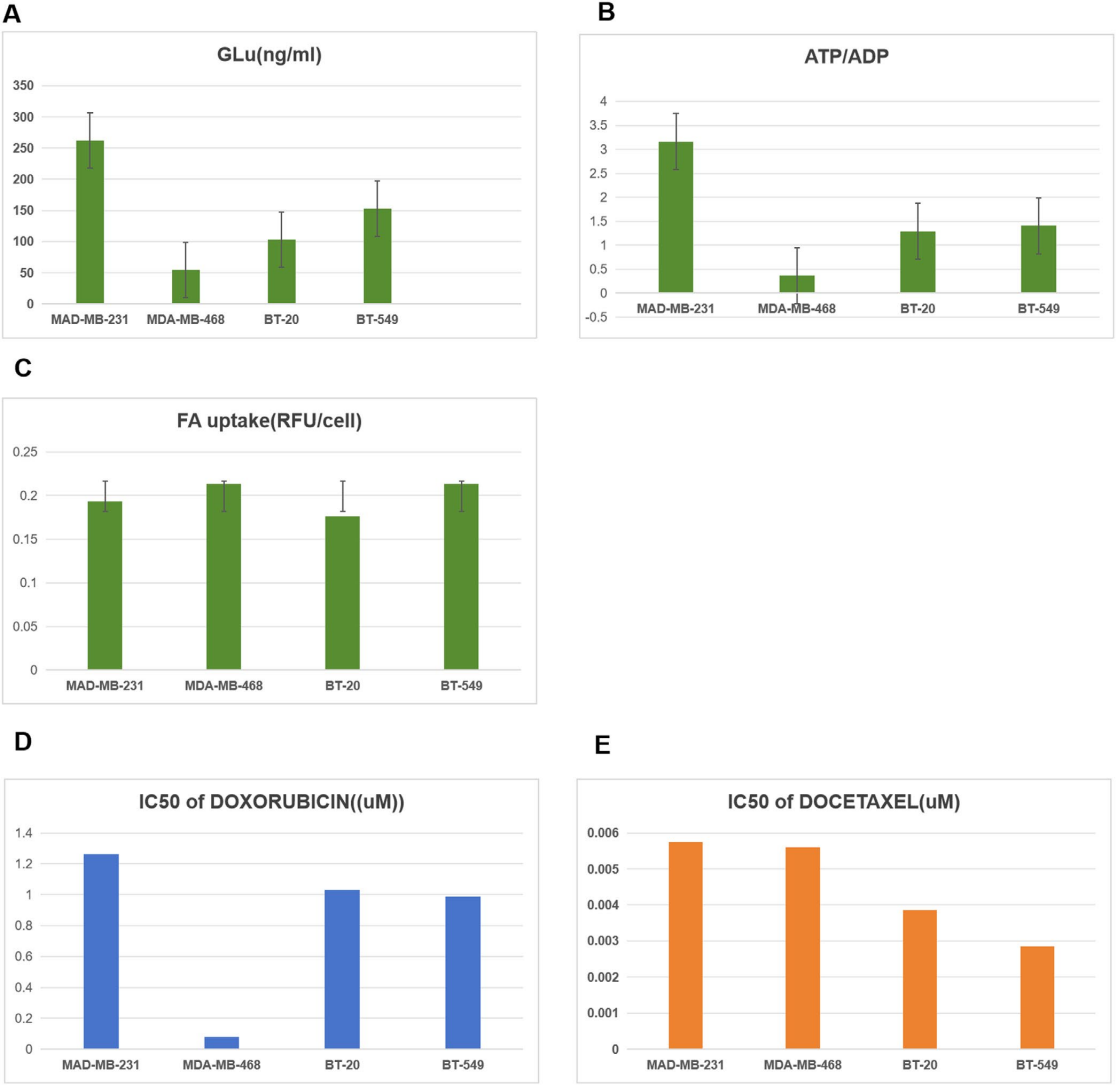
(A–C) The Glucose concentration (A), ATP/ADP values (B) and FA uptake rates (C) in MAD‐MB‐231, MDA‐MB‐468, BT‐20, and BT‐549 cells. (D, E) The IC50 values of Doxorubicin (D) and Docetaxel (E) among MAD‐MB‐231, MDA‐MB‐468, BT‐20, and BT‐549.

We examined the concentration of glucose (Figure 10A), ATP/ADP (Figure 10B) and free fatty acid uptake (Figure 10C) among MAD‐MB‐231, MDA‐MB‐468, BT‐20, and BT‐549 cells. The measurements of glucose and ATP/ADP were used to assess the glycolytic capacity of the cells, while the measurements of FA uptake were used to evaluate the cells' demand for fatty acids. The results indicated no significant differences in the levels of glucose and FA uptake across these cell lines. However, the ATP/ADP ratio in MAD‐MB‐231 cells was higher than in the other three cell lines, suggesting a potentially greater glycolytic activity in MAD‐MB‐231 cells.

Based on GDSC datasets, the IC50 values of these cell lines against doxorubicin and docetaxel were illustrated in Figure 10D,E, respectively. The results showed that the doxorubicin IC50 value for MAD‐MB‐231 cells was significantly higher than for the other cell lines, aligning with the predictions from MODIN. MODIN forecasted that MS_2 cells, characterised by enhanced carbohydrate and nucleotide metabolism, exhibit increased resistance to these drugs.

## 4 Discussion

In this study, we performed the consensus clustering of 52 metabolic pathways among TNBC patients and delineated four TNBC subtypes: MS_1 (glycosphingolipid biosynthesis‐enriched), MS_2 (hyperactive carbohydrate/nucleotide metabolism), MS_3 (pan‐metabolic activation), and MS_4 (metabolically suppressed).

In previous studies, the “Fudan Classification” system developed by Zhi‐Min Shao's stratified TNBC into four molecularly defined subtypes—immunomodulatory (IM), luminal androgen receptor (LAR), basal‐like immune‐suppressed (BLIS), and mesenchymal (MES)—establishing subtype‐specific therapeutic frameworks with clinical validation [18]. Metabolomic profiling by their work team further refined TNBC taxonomy into three clusters: the lipogenic subtype, marked by hyperactivation of fatty acid synthesis (ACACA, FASN) and cholesterol metabolism (HMGCR), showed therapeutic susceptibility to FASN inhibitors; the glycolytic subtype, driven by enhanced glycolysis (ENO2, LDHB) and nucleotide metabolism, exhibited potentiated immune checkpoint blockade efficacy following lactate dehydrogenase inhibition; and the mixed (MPS3) subtype displayed metabolic plasticity requiring mechanistic exploration [19].

Inspired by these insights, we developed MODIN, a deep learning framework integrating multi‐head attention mechanisms and pathway‐level multi‐omics features to predict drug sensitivity (IC50) in triple‐negative breast cancer (TNBC). MODIN advances prior computational models in two key aspects: (1) its capacity to resolve nonlinear interactions between cell‐line metabolic pathways (encoded as 3D feature maps via stacked CNNs) and drug structural fingerprints (represented by Rdkit‐derived sparse vectors), and (2) the stratification of TNBC into four metabolic subtypes (MS_1–MS_4) with distinct therapeutic vulnerabilities.

Based on MODIN prediction results, we found that the poor prognosis of MS_2 patients—characterised by hyperactive carbohydrate/nucleotide metabolism and suppressed lipid pathways—aligns with their resistance to doxorubicin and docetaxel. In vitro validation in MDA‐MB‐231 cells (high ATP/ADP ratio, Figure 6) corroborates this phenotype, implicating Warburg‐effect‐driven biosynthetic overload as a resistance driver. Mechanistically, excessive glucose/nucleotide flux in MS_2 depletes NADPH reserves [20], a key redox buffer, thereby exacerbating oxidative stress and compromising anthracycline efficacy. This suggests pre‐chemotherapy metabolic priming could sensitise MS_2 tumours: preclinical studies demonstrate that inhibiting glycolytic enzymes (e.g., HK2) or glutaminase restores NADPH pools and enhances doxorubicin response. Conversely, MS_3 tumours, despite pan‐metabolic activation, display paradoxical sensitivity to doxorubicin. We propose this stems from NADPH sink overload: robust fatty acid synthesis in MS_3 consumes NADPH to sustain lipid production, creating redox fragility. Doxorubicin‐induced AMPK suppression further disrupts acetyl‐CoA carboxylase (ACC)‐mediated lipogenesis, tipping the balance toward lethal metabolic crisis [21]. Clinically, combining doxorubicin with ACLY inhibitors (e.g., bempedoic acid) may amplify this vulnerability.

To bridge computational predictions and clinical utility, future work must integrate tumour microenvironment (TME) dynamics. Stromal‐immune interactions critically modulate drug response, yet MODIN currently focuses on tumour‐intrinsic metabolism. A new method, EV‐origin, can analyse RNA profiles in EVs from exLR‐seq data to infer their histocellular origin [22], helping researchers understand the dynamics of the tumour microenvironment and the mechanisms of tumour metastasis. In the context of pancreatic ductal adenocarcinoma (PDAC) research [23], exosome‐based exLR analysis has revealed that the dynamic shifts in cell types within the TME directly impact the metastatic potential of tumour cells and patient prognosis. The study by Li et al. optimised the sequencing strategy for human plasma exLRs (exLR‐seq), identifying multiple potential biomarkers useful for cancer diagnosis and the prediction of patient prognosis [24]. In various cancers, including breast cancer [25], colorectal cancer [26], and small cell lung cancer [27], numerous exLRs that exhibit differential expression between cancer and healthy controls have been reported, highlighting their potential as significant cancer biomarkers. Currently, the exoRBase database [28] and its updated version, exoRBase 2.0 [29], offer comprehensive data on exLRs present in human plasma‐derived exosomes, providing a rich resource for deepening our understanding of the role of exosomal RNA in the TME and its impact on tumour development and metastasis.

The findings of this study highlight the potential of metabolic subtyping and multi‐omics integration in addressing TNBC chemoresistance, yet several avenues warrant further exploration. First, while MODIN demonstrates robust predictive accuracy, enhancing its interpretability remains critical. Integrating visualisation tools such as Grad‐CAM with attention mechanisms could elucidate how specific metabolic pathways (e.g., nucleotide biosynthesis in MS_2) interact with drug structural motifs, offering mechanistic insights into resistance patterns. Second, the current reliance on static KEGG pathway enrichment may overlook dynamic metabolic adaptations. Future work should incorporate flux balance analysis and single‐cell metabolomics to map spatiotemporal metabolic heterogeneity within tumour microenvironments, particularly for MS_3 subtypes exhibiting pan‐metabolic activation.

To broaden applicability, extending MODIN to Warburg effect‐dependent cancers (e.g., pancreatic adenocarcinoma) or lipid metabolism‐driven malignancies (e.g., ovarian cancer) may establish a pan‐cancer metabolic vulnerability atlas. Collectively, these directions aim to bridge computational predictions with actionable therapeutic strategies, ultimately advancing TNBC's metabolic‐targeted management.

## 5 Conclusion

In this study, we have elucidated significant metabolic heterogeneity within Triple‐Negative.

This study has revealed significant metabolic heterogeneity within Triple‐Negative Breast Cancer (TNBC) by classifying patients into four distinct metabolic subtypes using GSVA analysis of metabolic pathways. The subtypes identified are: MS_1, characterised by increased lipogenic activity; MS_2, with elevated carbohydrate and nucleotide metabolism; MS_3, exhibiting broad activation of various metabolic pathways; and MS_4, showing suppressed metabolic activity.

Our novel Multiomics‐Driven Drug‐Cell Interaction Network (MODIN) demonstrated robust predictive capability for drug sensitivity in TNBC, with impressive performance metrics (MSE = 0.9762 and *R* ^2^ = 0.8254). Using MODIN, we uncovered significant disparities in drug sensitivity among the metabolic subtypes, particularly between MS_2 and MS_3. MS_3 patients showed significantly higher sensitivity to chemotherapy agents including doxorubicin and docetaxel, whereas MS_2 patients exhibited marked resistance to these drugs.

## Author Contributions

**Jingyuan Zhang:** data curation (lead), investigation (lead), methodology (lead), validation (equal), visualization (equal), writing – original draft (lead). **Xuejun Sun:** funding acquisition (lead), resources (lead), supervision (lead), writing – review and editing (lead).

## Consent

The authors have nothing to report.

## Conflicts of Interest

The authors declare no conflicts of interest.

## Data Availability

The data that support the findings of this study are available on request from the corresponding author. The data are not publicly available due to privacy or ethical restrictions.
